# Automated image curation in diabetic retinopathy screening using deep learning

**DOI:** 10.1038/s41598-022-15491-1

**Published:** 2022-07-01

**Authors:** Paul Nderitu, Joan M. Nunez do Rio, Ms Laura Webster, Samantha S. Mann, David Hopkins, M. Jorge Cardoso, Marc Modat, Christos Bergeles, Timothy L. Jackson

**Affiliations:** 1 Section of Ophthalmology, King’s College London, London, UK; 2 King’s Ophthalmology Research Unit, King’s College Hospital, London, UK; 3 South East London Diabetic Eye Screening Programme, Guy’s and St Thomas’ Foundation Trust, London, UK; 4 Department of Ophthalmology, Guy’s and St Thomas’ Foundation Trust, London, UK; 5 Department of Diabetes, School of Life Course Sciences, King’s College London, London, UK; 6 Institute of Diabetes, Endocrinology and Obesity, King’s Health Partners, London, UK; 7 School of Biomedical Engineering & Imaging Sciences, King’s College London, London, UK

**Keywords:** Retinal diseases, Medical imaging

## Abstract

Diabetic retinopathy (DR) screening images are heterogeneous and contain undesirable non-retinal, incorrect field and ungradable samples which require curation, a laborious task to perform manually. We developed and validated single and multi-output laterality, retinal presence, retinal field and gradability classification deep learning (DL) models for automated curation. The internal dataset comprised of 7743 images from DR screening (UK) with 1479 external test images (Portugal and Paraguay). Internal vs external multi-output laterality AUROC were right (0.994 vs 0.905), left (0.994 vs 0.911) and unidentifiable (0.996 vs 0.680). Retinal presence AUROC were (1.000 vs 1.000). Retinal field AUROC were macula (0.994 vs 0.955), nasal (0.995 vs 0.962) and other retinal field (0.997 vs 0.944). Gradability AUROC were (0.985 vs 0.918). DL effectively detects laterality, retinal presence, retinal field and gradability of DR screening images with generalisation between centres and populations. DL models could be used for automated image curation within DR screening.

## Introduction

Diabetes mellitus (DM) affects 463 million people worldwide, with the prevalence estimated to rise to 700 million by 2045^1^. Type 2 DM is the most common subtype affecting 90% of people with diabetes^2^. Diabetic retinopathy (DR) affects 30% of type 2 and 56% of type 1 diabetics^3^ and is a leading cause of acquired vision loss in working age adults^2,4^. Globally, DR is the fifth most common cause of blindness and the only one with an increased age-standardised prevalence between 1990 and 2020^5^.

DR screening using retinal photography aids in the early identification of sight-threatening DR (STDR), facilitating prompt referral and treatment which can reduce the risk of moderate visual loss by up to 50%^6^. In the UK, the Diabetic Eye Screening Programme (DESP) has been credited, in part, for the significant reduction in DR-associated sight-impairment^7^. However, the DESP is tremendously capital and labour intensive, costing more than £85 million per year in the England alone^8^. Given the increasing prevalence of DM and resource intense nature of DR screening, there has been substantial interest in automated retina image analysis systems (ARIAS), especially using deep learning (DL) networks, due to their impressive performance in DR classification^9–12^.

However, images must meet ARIAS specifications and quality requirements prior to analysis. In real-world DR screening programmes, large volumes of acquired images are affected by various factors including; capture technique (*defocused, over/under exposed, artefacts*), patient characteristics (*limited pupil dilation, motion blur, media opacities*) and other issues (*non-protocol retinal fields and miscellaneous images*)^13^. In the UK DESP, it is also customary to capture anterior segment views in patients who have co-pathology that affects the ability to take adequate retinal images (*e.g., dense cataracts*)^14^. The current curation process involves human assessment of image suitability prior to DR grading. With over 13 million images per year generated by the UK DESP, manual image curation is not a scalable solution. Therefore comprehensive, automated image curation systems are required and critically important to ensuring unsuitable images are excluded prior to manual or ARIAS-enabled DR grading as part of scalable clinical deployments^13,15^. Automated image curation systems could also be useful at the point of capture by providing real-time feedback to camera operators which can reduce the incidence of low-quality images by up to 70%^16,17^. Finally, automated curation models could be beneficial for research by identifying suitable images from large, open-access datasets which often have variable quality images.

**Figure 1:**
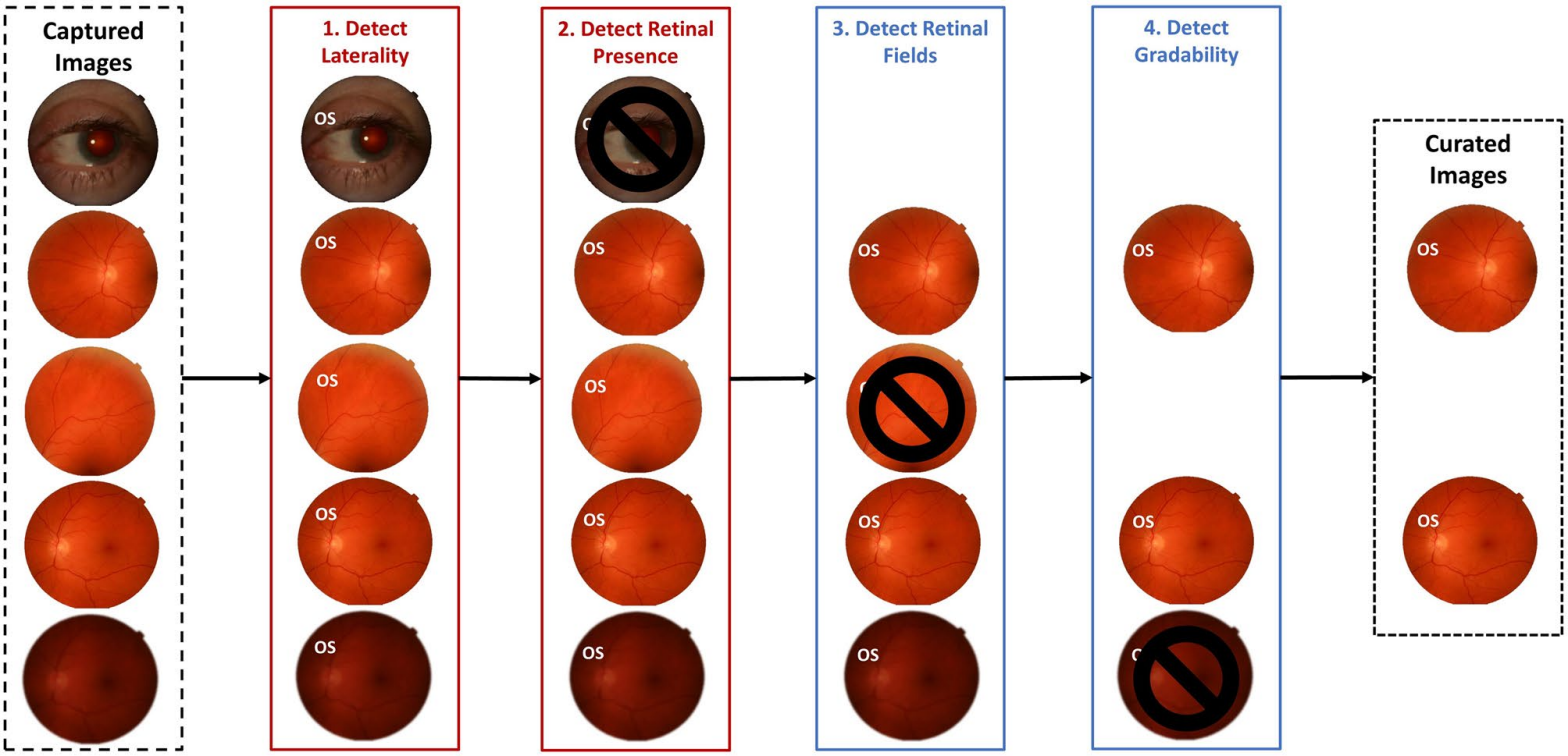
Automated image curation criteria. Automated image curation requires the detection of (1) laterality, (2) retinal presence (*retinal vs non-retinal images*), (3) retinal field (*macula vs nasal vs other retinal fields*) and (4) gradability which allows for the selection of gradable, 2-field retinal images of identifiable laterality for manual or automated DR grading.

Automating the curation of images captured during routine DR screening requires that systems identify four important characteristics: (1) laterality, (2) retinal presence (*if images are retinal or non-retinal which includes anterior eye images*), (3) retinal field (*macula vs nasal vs other retinal fields*) and (4) gradability (Fig. 1). Previous studies have explored the development of laterality^18–25^, retinal field^18,22,23^ and gradability^9,12,17,18,24–27^ detection systems largely in isolation, with limited research^18^ addressing the curation tasks in combination. Additionally, prior approaches required hand-crafted image pre-processing including feature extraction^28–33^, object detection^34,35^ or segmentation^36–39^. Finally, prior studies do not adequately describe patient characteristics^19–21,23–27^, assess for model bias^9,12,17,18^ or perform external validation^17,19,21,25^. It is also unclear which image features are key drivers of model predictions for the curation tasks. To the best of our knowledge, comprehensive automated curation systems for concurrent laterality, retinal presence, retinal field and gradability detection have not been reported.

We aim to develop and validate single and multi-output DL networks that classify four image characteristics: laterality, retinal presence, retinal field and gradability for automated image curation using routinely captured images from the large, longitudinal, ethnically diverse South-East London DESP (SEL-DESP). We aim to explore model performance parity by stratifying results by demographic characteristics (*age, sex, and ethnicity*). Finally, image features which drive model predictions will be evaluated using integrated gradient pixel attribution maps for each of the curation tasks.

## Results

**Figure 2:**
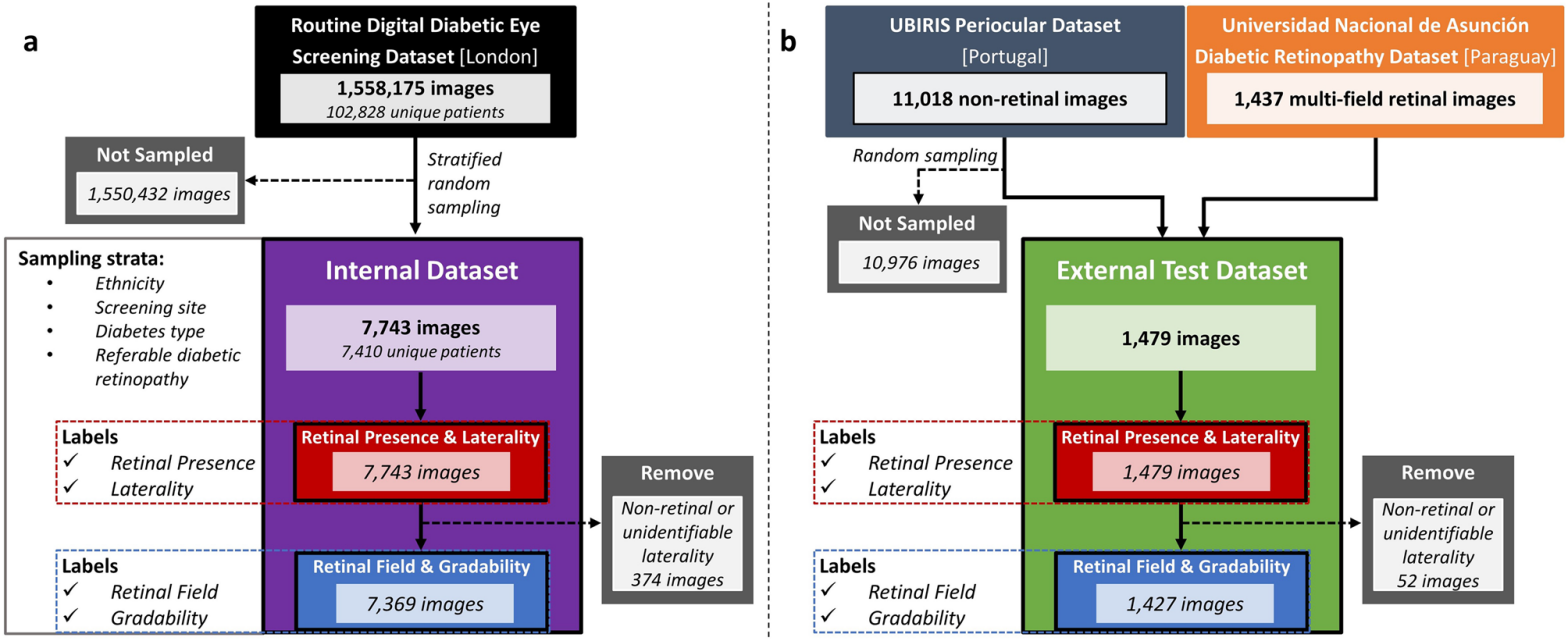
Study dataset flow chart.

The internal dataset was used for model development and internal testing. All 7743 images were used for laterality and retinal presence models, whilst 7369 images were used for retinal field and gradability models (*after removing 374 non-retinal and unidentifiable laterality images*). The external laterality and retinal presence model test set contained 1479 images, of which 1427 images were used for retinal field and gradability model testing after the removal of 52 non-retinal or unidentifiable laterality images (Fig. 2). Participant characteristics were mean (± standard deviation) age 63 ± 5 years, male 53%; type 2 diabetes 94%; mean diabetes duration 9 ± 8 years and STDR 4.1% for the routine digital diabetic eye screening dataset. These characteristics were matched following proportional sampling and splitting into training, tuning, and internal test datasets (Table 1) as were the contributions from individual DR screening sites (Supplementary Fig. S1). Image specifications for the internal and external datasets are shown in Supplementary Table S2.

**Table 1:** South-east London routine diabetic eye screening dataset characteristics.

Variable		Routine digital diabetic eye screening dataset N = 1,558,175		Laterality and retinal presence dataset N = 7,743				Retinal field and gradability dataset N = 7,369		
				Train (70%) N = 5446	Validation (10%) N = 756	Internal test set (20%) N = 1541		Train (70%) N = 5193	Validation (10%) N = 710	Internal test set (20%) N = 1466
		N (%) or mean (s.d.)		N (%) or mean (s.d.)				N (%) or mean (s.d.)		
Age (years)		63 (15)	Representative proportional random sampling	63 (15)	63 (15)	62 (15)	Non-retinal and unidentifiable laterality images removed (N = 374)	62 (15)	63 (14)	62 (15)
Sex	Male	832,092 (53.4)		2533 (53.5)	389 (48.5)	832 (54.0)		2793 (53.8)	358 (50.4)	807 (55.0)
Ethnicity	White	779,971 (50.1)		2739 (50.3)	378 (50.0)	772 (50.1)		2615 (50.4)	355 (50.0)	749 (51.1)
	Black	462,143 (29.7)		1627 (29.9)	212 (28.0)	462 (30.0)		1535 (29.6)	211 (29.7)	428 (29.2)
	South Asian^a^	100,861 (6.5)		354 (6.5)	45 (6.0)	99 6.4)		339 (6.5)	37 (5.2)	96 (6.5)
	Other Asian^b^	101,296 (6.5)		342 (6.3)	60 (7.9)	103 (6.7)		328 (6.3)	56 (7.9)	97 (6.6)
	Mixed	41,038 (2.6)		148 (2.7)	13 (1.7)	37 (2.4)		140 (2.7)	16 (2.3)	35 (2.4)
	Other^c^	40,744 (2.6)		136 (2.5)	25 (3.3)	36 (2.3)		134 (2.6)	18 (2.5)	32 (2.2)
	Not specified	32,122 (2.1)		100 (1.8)	23 (3.0)	32 (2.1)		102 (2.0)	17 (2.4)	29 (2.0)
Diabetes type	Type 2	1,456,971 (93.5)		5122 (94.1)	709 (93.8)	1445 (93.8)		4868 (93.7)	667 (93.9)	1380 (94.1)
	Type 1	95,728 (6.1)		317 (5.8)	46 (6.1)	91 (5.9)		317 (6.1)	41 (5.8)	84 (5.7)
	Other	1842 (0.1)		2 (< 0.1)	1 (0.1)	2 (0.1)		3 (0.1)	1 (0.1)	0 (0)
	Not Specified	3634 (0.2)		5 (< 0.1)	0 (0)	3 (0.2)		5 (0.1)	1 (0.1)	2 (0.1)
Diabetes duration (years)		9 (8)^1^		9 (8)^2^	9 (7)^3^	9 (8)^4^		9 (8)^5^	9 (8)^6^	9 (8)^7^
DR grade	No STDR	1,487,832 (95.5)		5213 (95.7)	718 (95.0)	1474 (95.7)		4962 (95.6)	691 (97.3)	1412 (96.3)
	STDR	64,125 (4.1)		215 (3.9)	33 (4.4)	64 (4.2)		223 (4.3)	19 (2.7)	54 (3.7)
	Not Specified	5633 (0.4)		18 (0.3)	5 (0.7)	3 (0.2)		8 (0.2)	0 (0)	0 (0)
Laterality	Right	–		2716 (49.9)	369 (48.8)	723 (46.9)		2561 (49.3)	330 (46.5)	721 (49.2)
	Left	–		2726 (50.1)	385 (50.9)	816 (53.0)		2632 (50.7)	380 (53.5)	745 (50.8)
	Unidentifiable	–		4 (< 0.1)	2 (0.3)	2 (0.1)		–	–	–
Retinal presence	Non-retinal	–		256 (4.7)	34 (4.5)	84 (5.5)		–	–	–
	Retinal	–		5190 (95.3)	722 (95.5)	1457 (94.5)		5193 (100)	710 (100)	1466 (100)
Retinal field	Macula	–		–	–	–		2379 (45.8)	322 (45.4)	673 (45.9)
	Nasal	–		–	–	–		2350 (45.3)	335 (47.2)	653 (44.5)
	Other retinal field	–		–	–	–		464 (8.9)	53 (7.5)	140 (9.5)
Gradability	Ungradable	–		–	–	–		872 (16.8)	108 (15.2)	257 (17.5)
	Gradable	–		–	–	–		4321 (83.2)	602 (84.8)	1209 (82.5)

^a^Includes Indian, Bangladeshi, and Pakistani ethnic backgrounds, ^b^Includes any other asian background or Chinese, ^c^Includes any other ethnic group or Arab, Missing values: ^1^5,665, ^2^13, ^3^2, ^4^6, ^5^17, ^6^2, ^7^2, N: Images, s.d. Standard deviation, DR: Diabetic retinopathy, STDR: Sight-threatening diabetic retinopathy.

Of the 7,743 internal dataset images, 50.7% were from right eyes and 4.8% were non-retinal. Only images which did not have any discernible anterior eye or retinal features were labelled as having an unidentifiable laterality (0.1%, 8 images). After removing non-retinal and unidentifiable laterality images, 7,369 retinal images remained of which, 91.1% were from macula or nasal fields, and 83.2% were gradable. The proportions of these curation characteristics were largely concordant following the partitioning of the dataset into training, tuning, and internal test sets. No participant characteristics were available for the external test datasets, however, internal and external datasets differed significantly with regards to STDR (4.2% vs 48.7%), macula (45.9% vs 74.2%), nasal (44.5% vs 11%) and ungradable (17.5% vs 28.6%) image proportions (Table 1 and Supplementary Table S3).

### Automated image curation model performance

#### Single-output model approach

##### Internal test

**Figure 3:**
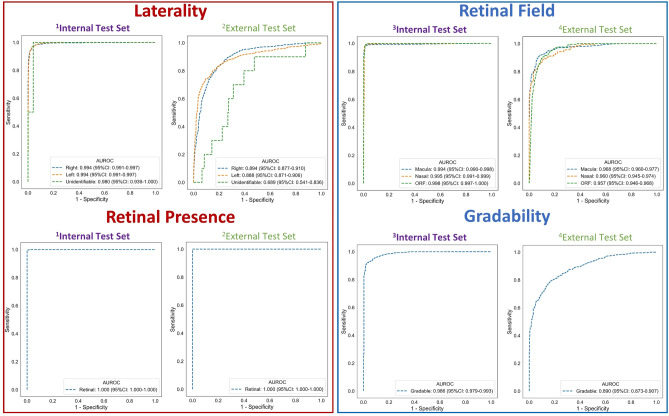
Single-output model receiver operating characteristic curves. ^1^Test set size = 1541 images, ^2^Test set size = 1479 images, ^3^Test set size = 1466 images, ^4^Test set size = 1427 images. AUROC: area-under-the receiver operating characteristic curve, ORF: other retinal field.

Laterality area-under-the receiver operating characteristic (AUROC) for right, left and unidentifiable classes were 0.994 (95% Confidence Interval: 0.991–0.997), 0.994 (0.991–0.997) and 0.980 (0.939–1.000), respectively. Retinal presence AUROC was 1.000 (1.000–1.000) for the retinal class. Retinal field AUROC for macula, nasal and other retinal field classes were 0.994 (0.990–0.998), 0.995 (0.991–0.999) and 0.998 (0.997–1.000) respectively. Gradability AUROC was 0.986 (0.979–0.993) for the gradable class (Fig. 3).

##### External test

Laterality AUROC for right, left and unidentifiable classes were 0.894 (0.877–0.910), 0.888 (0.871–0.906) and 0.689 (0.541–0.836), respectively. Retinal presence AUROC was 1.000 (1.000–1.000) for the retinal class. Retinal field model AUROC for macula, nasal, and other retinal field classes were 0.968 (0.960–0.977), 0.960 (0.945–0.974) and 0.957 (0.946–0.968) respectively. Gradability model AUROC was 0.890 (0.873–0.907) for the gradable class (Fig. 3).

#### Multi-output model approach

##### Internal test

**Figure 4:**
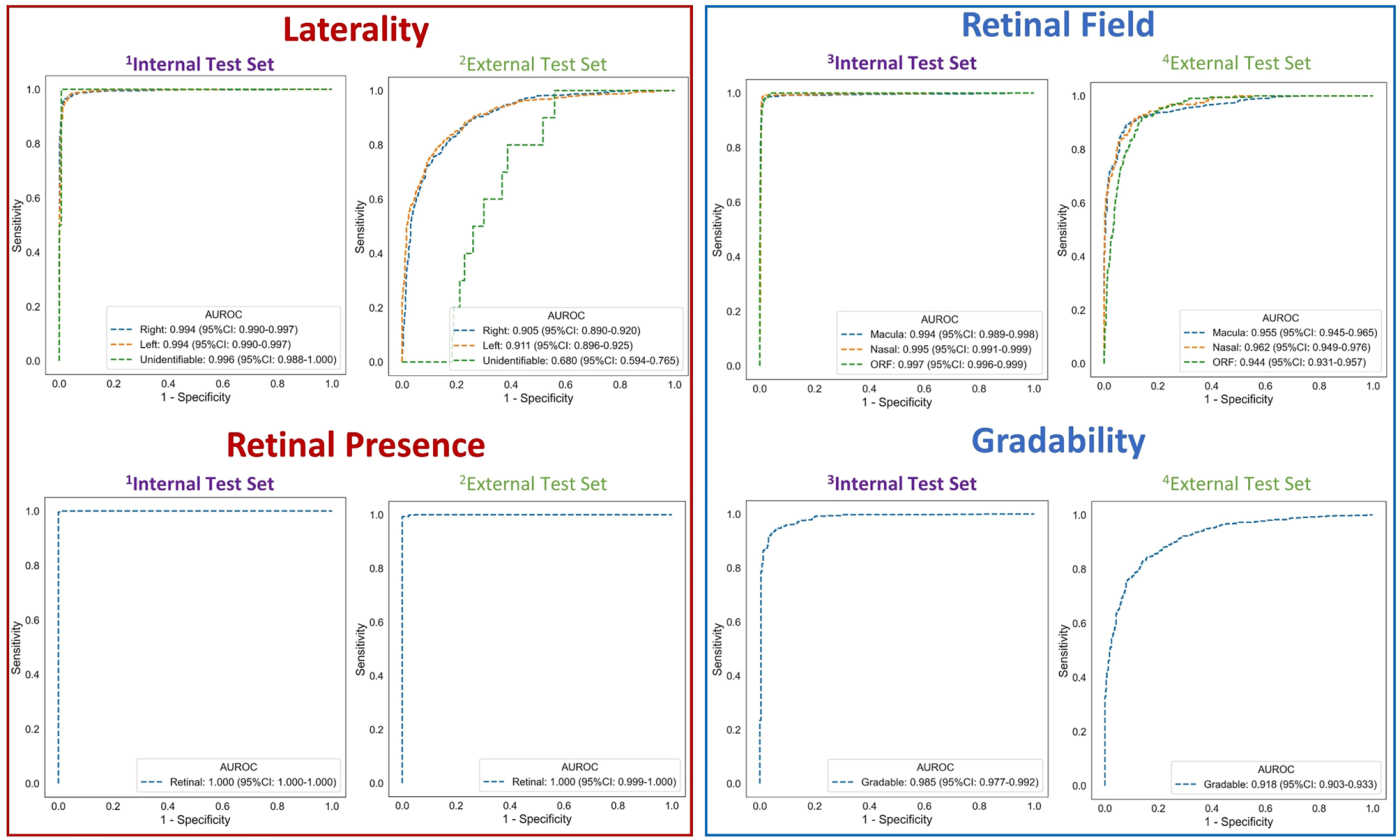
Multi-output model receiver operating characteristic curves. ^1^Test set size = 1541 images, ^2^Test set size = 1479 images, ^3^Test set size = 1466 images, ^4^Test set size = 1427 images. AUROC: area-under-the receiver operating characteristic curve, ORF: other retinal field.

Laterality and retinal presence AUROC were 0.994 (0.990–0.997, *p* vs single-output model: 0.739), 0.994 (0.990–0.997, *p*: 0.555) and 0.996 (0.988–1.000, *p*: 0.494) for right, left and unidentifiable classes respectively, with an AUROC of 1.000 (1.000–1.000, *p*: 0.739) for the retinal class. Retinal field and gradability AUROC were 0.994 (0.989–0.998, *p*: 0.497), 0.995 (0.991–0.999, *p*: 0.632) and 0.997 (0.996–0.999, *p*: 0.075) for macula, nasal, and other retinal field classes respectively with an AUROC of 0.985 (0.977–0.992, *p*: 0.361) for the gradable class (Fig. 4). To simulate real-world use and assess for error propagation from applying models sequentially, we used laterality labels from the laterality and retinal presence DL multi-output model to flip left eye images to right eye orientation instead of using the ophthalmologist defined ground truth laterality label. Retinal field and gradability AUROC remained largely the same at 0.992 (0.988–0.997), 0.991 (0.986–0.995) and 0.996 (0.994–0.998) for macula, nasal, and other retinal field classes respectively and 0.983 (0.976–0.992) for the gradable class.

##### External test

Laterality and retinal presence AUROC were 0.905 (0.890–0.920, *p* vs single-output model: 0.07), 0.911 (0.896–0.925, *p*: 0.002), 0.680 (0.594–0.765, *p*: 0.897) for right, left and unidentifiable classes respectively, with an AUROC of 1.000 (0.999–1.000, *p*: 0.271) for the retinal class. Retinal field and gradability AUROC were 0.955 (0.945–0.965, *p*: < 0.001), 0.962 (0.949–0.976, *p*: 0.549), 0.944 (0.931–0.957, *p*: 0.012) for macula, nasal, and other retinal field classes respectively, with an AUROC of 0.918 (0.903–0.933, *p*: < 0.001) for the gradable class (Fig. 4). Using laterality model derived labels to horizontally flip left eye images, retinal field and gradability AUROC were 0.914 (0.899–0.929), 0.936 (0.912–0.961) and 0.920 (0.904–0.936) for macula, nasal, and other retinal field classes respectively and 0.896 (0.880–0.913) for the gradable class.

### Multi-output model internal test set performance stratified by demographic characteristics

Laterality and retinal presence multi-output model sensitivity on the internal test dataset were comparable after stratification by age group, sex, and ethnicity with the exception of laterality sensitivity which was marginally reduced for the ≥ 80 year age group (0.88 vs 0.96, Supplementary Table  S4). For some strata, due to the limited number of non-retinal samples, retinal presence specificity could not be estimated with a high degree of confidence. However, for subgroups with sufficient negative cases, retinal presence specificity did not vary significantly.

Retinal field and gradability sensitivity and specificity were similar between age, sex, and ethnicity groups for the multi-output model with the exception of gradability sensitivity/specificity for the ≥ 80 year age group (0.90/0.78 vs 0.97/0.86) and mixed ethnicity groups (0.90/0.67 vs 0.97/0.86). In addition, gradability specificity for the Black ethnicity group was also marginally reduced (0.80 vs 0.86), but it should be noted that due to the limited ungradable images in some subgroups, the gradability specificity confidence intervals were relatively broad.

### Multi-output model laterality and gradability internal test set performance stratified by retinal field

Laterality sensitivity and specificity for the multi-output model on the internal test dataset were ≥ 97% for macula and nasal fields and 93% for other retinal fields (Supplementary Table S5).

Gradability sensitivity for the multi-output model were high for macula and nasal fields (≥ 96%) but the specificity was lower at 76% and 69% respectively. The model had a high gradability specificity (98%) for other retinal fields indicating accurate detection these images as ungradable.

### Pixel attribution maps

**Figure 5:**
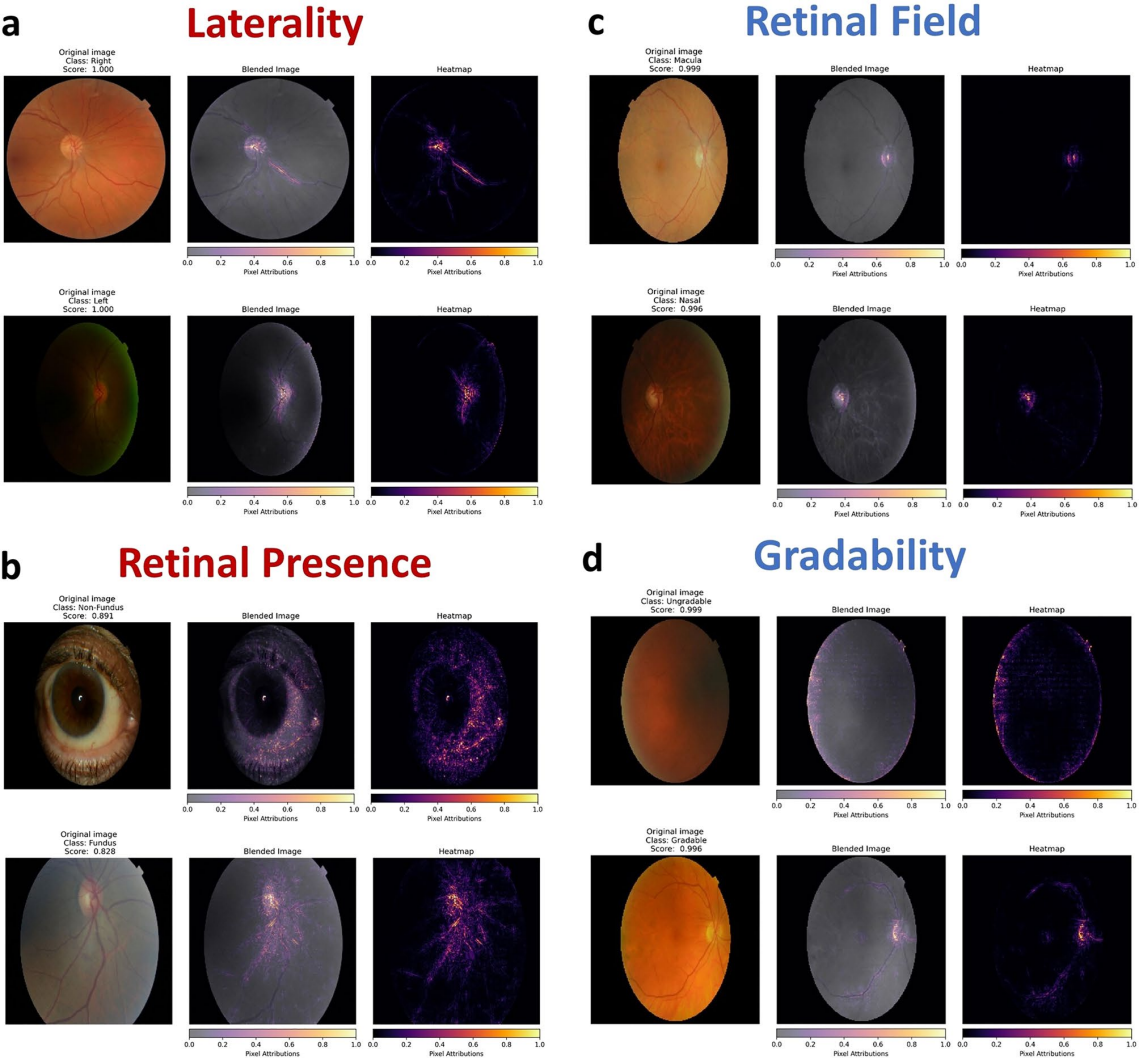
Internal test single-output model pixel attribution maps. Integrated gradients pixel attributions: all models highlight the optic cup/disc within retinal images, especially model c. Models a, b and d also highlight the retinal vessels to varying degrees. Model b (*non-retinal image*) highlights the caruncle, lower tear meniscus, iris striations, conjunctival vessels, and corneal reflection. Model attributions relative to the true positive class in each image.

Single-output model integrated gradient pixel attribution map examples for the four curation tasks are shown in Fig. 5 (*internal test*) and Supplementary Fig. S5 (*external test*). Attribution maps for laterality detection demonstrate that the optic cup/disc and proximal retinal vasculature are the significant driver features amongst retinal images (Fig. 5a). Similarly, retinal images are distinguished by the presence of the optic cup/disc and vascular tree, whilst iris striations, conjunctival vessels, corneal reflections, caruncle, and tear meniscus are highlighted as important features for non-retinal (*anterior segment*) image identification (Fig. 5b). The optic cup/disc was also the main feature which was determinant to macula or nasal field predictions (Fig. 5c). Finally, the optic cup/disc and vascular arcades were also important to the detection of image gradability, with the image edge highlighted in a fairly featureless ungradable image (Fig. 5d).

## Discussion

**Figure 6:**
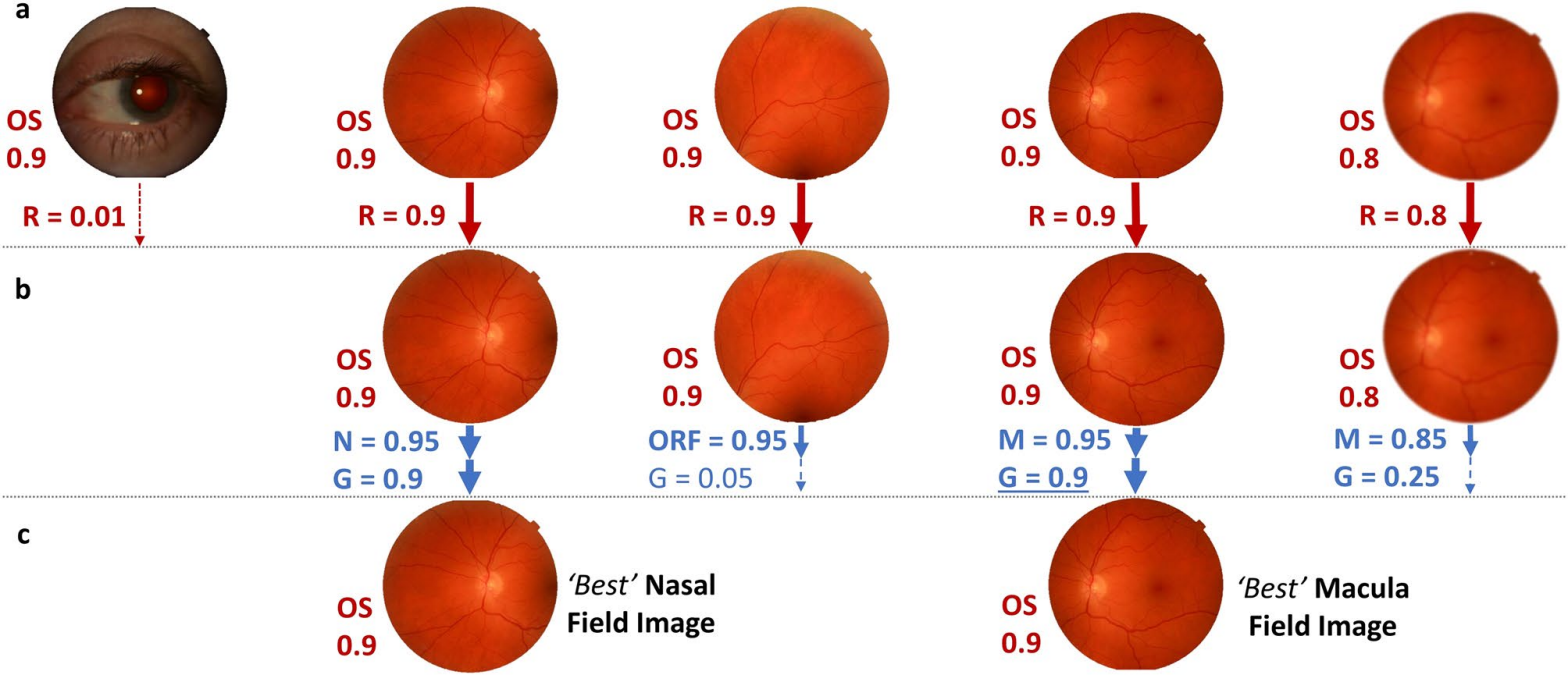
Proposed curation workflow. (**a**) Images get predictions for laterality and retinal presence (*values indicate model predictions between 0 and 1*) allowing for the exclusion of non-retinal images (*e.g., anterior segment*). (**b**) Images obtain retinal field and gradability predictions which allows for the exclusion of other retinal field images and for the selection of gradable images from macula or nasal fields by selecting the image with the highest gradable score (*underlined*). (**c**) The ‘best’ macula and nasal field with an identifiable laterality are then selected; these gradable, 2-field images are then suitable for subsequent manual or automated diabetic retinopathy grading. R: Retinal presence, OS: Left eye, N: Nasal, M: Macula, ORF: Other retinal field, G: Gradability.

To the best of our knowledge, this is the first four-in-one (*laterality, retinal presence, retinal field and gradability*) comprehensive automated DL curation system for images captured during routine DR screening. We developed two approaches for the automation of a four-label image curation system based using four sequential single-output models or two multi-output sequential models, respectively. Models were validated in two datasets, an internal test set containing images sampled from a large, longitudinal, ethnically diverse, multisite DR screening programme in the UK, and an open-access external dataset containing images from a hospital-based DR dataset from Paraguay and a periocular dataset from Portugal. Both single and multi-output approaches demonstrated excellent performance on all the specific curation tasks in the internal test dataset, which generalised well to the external test set despite its more challenging and heterogeneous images. Multi-output models outperformed single-output models in left and gradability classification in the external dataset but with reduced macula and other retinal field detection. These results suggest that for some co-trained tasks, there may be performance and generalisation advantages to using multi-output DL models, but this may come at the cost of reduced performance on other tasks. Additionally, however, multi-output models can simplify training and reduce inference time compared to using a multitude of individual single-output models. Figure 6 shows a proposed workflow for automated image curation whereby image laterality and retinal presence are initially identified, simultaneously in the case of multi-output models. Non-retinal images which had an identifiable laterality were from the anterior eye. Identification of anterior eye images may be useful given recent work suggesting DL models can detect disease and systemic biomarkers using these images^40^. After removing non-retinal images and those with unidentifiable laterality, retinal field and gradability classification is performed (*simultaneously in the case of multi-output models*), allowing for the selection of a pair of gradable macula and nasal images for onward manual or automated 2-field DR grading. Modelling approaches and curation systems used in this study could also be applied to other clinical pathways reliant on colour photos where there is variability in imaged fields and gradability.

Laterality (*right and left*) internal/external test performance was competitive when compared to previous DL based approaches (*AUROC*: 1.000^18^, 0.995^20^, 0.989^25^, 0.976^22^, 0.920^19^, *accuracy:* 98.98%^21^, ≥ 98.6^23^, *sensitivity*: left 90.1% and right 91.6%^24^) despite the laterality model classifying both multifield retinal images and non-retinal (*e.g., anterior eye*) images, whereas prior approaches focused on macula or nasal field images alone. However, the laterality model also had excellent classification performance when stratified by retinal field (Supplementary Table S5). A reported DL model trained to classify laterality in anterior segment images alone achieved an AUROC of 0.998^41^. The classification of unidentifiable laterality images was excellent in the internal test set but reduced in the external dataset. This may be due to differences between datasets, with significantly more cases of advanced DR with obscuring retinal haemorrhages in the external test set. Reduced model performance may also be due to the limited number of examples were the laterality was unidentifiable (*6 in the development dataset*) which subsequently impacted generalisation to the external dataset. Images with misidentified laterality in the external dataset were all from other retinal fields, hence would likely be detected by retinal field or gradability models and subsequently excluded. Therefore, laterality misclassification minimally impacts downline image selection for subsequent DR screening.

Prior feature-based classification methods reported variable success in identifying non-retinal images (*accuracy*: 85.00%^42^, 99.54%^43^). In this study, retinal images were distinguished from non-retinal images extremely well in both the internal and external test sets using DL. This is very reassuring because these models would effectively safeguard against the selection of non-retinal images for downstream DR grading, which would otherwise be detrimental to ARIAS STDR detection^13^.

Few studies have evaluated the detection of macula, nasal, and other retinal fields simultaneously. One study used a U-Net optic disc semantic segmentation and rule-based classification of the predicted mask with an overall accuracy of 99.0%^23^. Other studies focused on macula and nasal field classification alone, with the prerequisite that images were gradable, and reported an AUROC of 1.000^18^ and 0.957^22^. Bellemo et al*.,* found model performance generalised well between different ethnicity groups in concordance with our findings^22^. Our retinal field model results, therefore, compare favourably to prior studies given the diversity of the development dataset which varied in imaging devices, locations, populations, and image quality.

Gradability definitions vary between studies and differ from current UK DR screening guidelines^14^, making it challenging to compare results. However, internal test set performance are on par with previous DL-based approaches for gradability classification, with reported AUROC of 0.987^18^, 0.980^9^, 0.947^25^, 0.986^26^, 0.934^17^, 0.914^27^, and reported ungradable sensitivity of 81.3%^24^ and 70.9%^12^. Gradability sensitivity was excellent with good specificity when stratified by retinal field (Supplementary Table S5). There was high gradability specificity for other retinal fields indicating the gradability model accurately detects these ungradable images. Differences in performance between the internal and external sets are likely due to higher STDR (49.8% vs 3.7%) and ungradable (28.6% vs 17.5%) images in the external dataset which were associated with significant higher levels of image obscuration (*e.g., from DR-associated vitreous haemorrhage or advanced cataract*). We evaluated real-world use by simulating the sequential application of the laterality model to obtain laterality labels which we use to horizontally flip left eye images for the retinal presence and gradability model. We found almost identical internal test performance and a minimal reduction in external test performance (*0.02–0.04 AUROC difference*) compared to using ophthalmologist defined laterality labels, indicating low error propagation from applying models sequentially.

Prior studies have not evaluated for image curation model performance parity with respect to age, sex, and ethnicity. It is important to examine for disparity in DL model outputs to ensure that systems deployed in clinical practice do not unduly introduce or reinforce biases^44^. Importantly, we ensured that our internal development and testing datasets were representative of the source DR screening population, to reduce the risk of introducing biases during model training. The automated curation models demonstrated equal sensitivity between groups for all curation tasks, with the exception of the ≥ 80 year age group. Reduced laterality and gradability sensitivity performance in this group may be explained by the potentially higher occurrence of cataract or other media opacities, which adversely affect image quality and subsequently degrade the clarity of image features used by models to classify laterality and gradability.

Similarly, few studies have evaluated image features which are the key drivers for curation model predictions. Jang et al. and Rim et al. analysed class activation maps and found that the optic disc and proximal retinal vasculature were important features for laterality classification, in agreement with our observations^21,23^. Uniquely, we also found that the optic disc and retinal vasculature have the greatest influence on model predictions for retinal presence, retinal field and gradability classification. This finding supports the strategy of using a multi-output model, given the shared features between the curation tasks. Attribution maps also reveal that models learned to use distinctive features (*conjunctival vessels, corneal reflections, tear meniscus, and caruncle*) to distinguish anterior eye from retinal images. Interestingly, despite significant differences in the imaged periocular area of the non-retinal external test set, attribution maps demonstrated that the retinal presence models utilized similar image features (*corneal reflections and conjunctival vessels*) to the internal test set to generate predictions for periocular images.

This study improves upon prior approaches in a several aspects. The study source dataset is a large, longitudinal, ethnically diverse, multisite DR screening programme, which therefore captures the variations that exist in participant demographics, screening sites, imaging techniques, and devices. The source DR screening dataset was proportionally sampled to ensure participant diversity was maintained, and the sampled dataset was reflective of the routine DR screening population. We described in detail key image, participant, and disease characteristics (*e.g., STDR*) for each step of model training, validation, and testing. Model performance was assessed with respect to important demographic characteristics to evaluate for discriminatory effects, a critical requirement for automated curation systems that would be deployed in heterogenous clinical populations^44^.

Study limitations are the lack of multiple graders or repeat grading which precluded the ability to assess inter/intra-grader performance or adjudication in cases of disagreement. In our prior study which developed curation DL models for handheld non-mydriatic retinal images from community-based DR screening, the intra-grader agreement was (Kappa) 0.78/0.94 with an inter-grader agreement of 0.59 for gradability in a challenging dataset, therefore, a similar or better level of agreement would be expected in this study given the use of mydriatic, desktop retinal imaging^45^. Further limitations are the limited development samples within the unidentifiable laterality class and lack of a single source external test dataset of routine DR screening multifield, and variable quality retinal and non-retinal images for additional validation. Although significant care was taken to proportionally sample images for model development with regard to important participant characteristics, imbalance in other attributes may remain and the relatively conservative sample size may not capture the full distribution of images which occurred within the whole source DR screening dataset.

Our results demonstrate that DL systems can be used for the comprehensive, automated curation of images captured during routine DR screening, with generalisation across populations and sites. Study approaches based on sequential classification DL models perform well despite significant differences in imaging devices, DR severity and DR screening protocols. Developed DL models could enable the automated curation of large image sets which are routinely captured within DR screening in support of downstream manual or ARIAS-driven DR grading. Study approaches for automated image curation are also of relevance to other clinical pathways with large, heterogeneous fundus image datasets. Future prospective clinical validation studies should evaluate the efficacy of automated image curation and subsequent effects on DR severity grading. Future studies should also evaluate if on-imaging-device feedback from developed automated image curation models improve the quality of images captured in routine DR screening and effects on subsequent STDR detection as well as performance of the curation models in non-DR screening datasets.

## Methods

This study was conducted in accordance with the tenets of the Declaration of Helsinki. UK Health Research Authority approval and a favourable ethical opinion from the UK East Midlands Leicester South Research Ethics Committee were attained prior to study commencement (20/EM/0250, 6/October/2020). The need for informed consent was waived by the favourable ethical opinion. Study data were anonymised prior to extraction, however, participants who previously objected to the use of their data for research were excluded.

### Internal dataset

Digital images taken between September 2013 to December 2019 across 27 different DR screening sites of SEL-DESP were eligible for inclusion. Images were captured after mydriasis (1% tropicamide) within hospitals and community settings, such as opticians. Characteristics data were collected including year of birth, sex, ethnicity, diabetes type, diabetes duration and final retinopathy/maculopathy grade. All screening procedures including image capture, DR grading and initial data collection, were performed by trained SEL-DESP graders as part of routine DR screening using established protocols^46^. STDR was defined as referable DR (R2 or R3A, *moderate or worse DR*) with or without referable maculopathy (M1) as per the UK national screening committee criteria^46^; retinopathy and maculopathy grading definitions are summarised in Supplementary Table S6. A total of 1,558,175 images from 102,828 patients attending routine DESP screening were extracted.

### Ground-truth creation

A representative random sample of 7,743 images (Fig. 2a) was manually labelled for laterality *(right, left, unidentifiable)* and retinal presence (*retinal, non-retinal [includes anterior eye and miscellaneous images]*). Images which had an identifiable laterality (right or left) and were retinal subsequently underwent labelling for retinal field (*macula, nasal, other retinal field*) and gradability (*gradable, ungradable*). Therefore, the retinal field/gradability dataset was a subset of the total study dataset and included retinal images of known laterality. All labelling was performed by an experienced ophthalmology fellow trained in DR grading. A custom labelling app (Supplementary Fig. S6) was created to ensure there was a consistent grading environment and to maximise the robustness of the labelling process. Definitions used for the creation of the ground truth labels are presented in detail in the Supplementary Information, with examples shown in Supplementary Fig. S7.

### External test dataset

An external dataset comprised of a composite of 1,479 images was created by combining two sources to overcome the lack of open-access datasets that include both non-retinal and multi-field retinal images. A sample of 42 non-retinal images from the UBIRIS periocular dataset (Portugal)^47^ were randomly selected to ensure that when combined with the 1,437 retinal images from Universidad Nacional de Asunción hospital DR dataset (Paraguay) (Fig. 2b), the percentage of non-retinal images (2.8%) was proportional to the internal dataset (4.8%) but with a degree of residual variation in order to construct a challenging test dataset^48^.

### Model development

The internal dataset was randomly split into 70% for training, 10% for validation and 20% for internal testing at the patient-level. All internal dataset images were used in laterality and retinal presence model development and testing. Thereafter, non-retinal images and those without an identifiable laterality were removed prior to the development and testing of retinal field and gradability models.

Patients included in the train/validation/internal testing sets differed between laterality/retinal presence and retinal field/gradability datasets. However, their characteristics were comparable, and representative of the source population as shown in Table 1. Four single-output DL models were developed which classified laterality, retinal presence, retinal field or gradability respectively. Two multi-output DL models were also developed which simultaneously identified laterality and retinal presence or retinal field and gradability. Multi-output models were grouped by laterality/retinal presence and retinal field/gradability tasks given the synergy between latter tasks (*i.e., a gradable image must be from a macula or nasal field*). Multi-output models are advantageous because of touted improvements in regularisation and generalisation with multi-task training^49^, and because at deployment, only two multi-output models are required for automated curation instead of four single-output models, which significantly reduces inference time.

EfficientNet-V1-B0 with ImageNet weight initialisation was used as the feature extractor (*encoder*), followed by an untrained, randomly initialised classification network with 3 × 3 depth-wise separable 2D convolutions^50^, batch normalisation^51^ and flattened feature layers prior to a final dropout layer^52^ and classification node. Using pre-determined optimal hyperparameters, models were trained with a batch size of 32 for a maximum of 60 epochs with an exponentially decaying learning rate after 2 epochs, with early stopping criteria when there was a 3-epoch plateau in the validation set AUROC (*single-output models*) or loss (*multi-output models*). EfficientNet model weights were frozen until validation set metrics reached a plateau, then unfrozen until either the maximum epoch or early termination conditions were met. The model with the maximum validation set AUROC (*single-output models*) or minimum loss (*multi-output models*) during training were selected for testing. Models were developed on × 2 P6000 NVIDIA GPUs using python (v3.8.2) and Tensorflow (v2.5.0) open-source libraries. Image pre-processing and additional model development details are discussed in the Supplementary Information and the multi-output model architecture is shown in Supplementary Fig. S8.

### Pixel attribution maps

Integrated gradients, an axiomatic feature attribution method, were used to ascertain image pixels which were most influential to model predictions^53^. A ‘heatmap’ of per pixel attributions relative to the target class were computed and displayed both in isolation and overlayed on a grayscale version of the original image, allowing for a subjective comparison of pixel attributions and image features. Single-output model integrated gradient pixel attribution map examples for the four curation tasks are shown in Fig. 5 (*internal test*) and Supplementary Fig. S5 (*external test*).

### Statistical analysis

Receiver operating characteristic (ROC) curves and AUROC were used to summarise model performance, with multi-output ROC/AUROC computed using a one-vs-all strategy. Mid-operating point (*threshold 0.5*) and largest prediction index (*argmax function*) for binary and multiclass labels respectively were used to compute multi-output model sensitivity and specificity stratified by age, sex, and ethnicity to assess for performance equivalence within subgroups. Confidence intervals for the AUROC and sensitivity/specificity were estimated using the Delong^54^ and exact Clopper-Pearson^55^ methods, respectively. Delong’s test was used to compare single and multi-output AUROC in the internal and external test datasets with a significance level of *p* ≤ 0.05^54^. Dataset characteristics are reported as means and standard deviations for continuous variables or counts and proportions for categorical variables with analyses performed using SPSS (v27), IBM, Chicago, Illinois and statsmodels (v0.12.2) open-source python library.

## Supplementary Information

Supplementary Information.

## Data Availability

The external test datasets are freely accessible, and links are provided in Supplementary Table S2 with associated ground truth labels from this study available at https://github.com/pnderitu/DUK_Automated_Curation.git.

## Code availability

The code used to train, tune and test single and multi-output Tensorflow models are available from https://github.com/pnderitu/DUK_Automated_Curation.git.
