# Supplementary material for: Automated image curation in diabetic retinopathy screening using deep learning
Source: Sci Rep. 2022 Jul 1;12:11196. doi: 10.1038/s41598-022-15491-1 (PMC9249740; doi:10.1038/s41598-022-15491-1)
Supplement: Supplementary file 1 — Supplementary Information. [file 41598_2022_15491_MOESM1_ESM.docx]

**Supplementary Materials**

Automated Image Curation in Diabetic Retinopathy Screening using Deep Learning

Paul Nderitu, FRCOphth^1,2*^; Joan M. Nunez do Rio, PhD^1^; Ms Laura Webster^3^; Samantha S. Mann, MD^3,4^; David Hopkins, PhD^5,6^; M. Jorge Cardoso, PhD^7^; Marc Modat, PhD^7^; Christos Bergeles, PhD^7^; Tim Jackson, PhD^1,2^.

^1^Section of Ophthalmology, King’s College London, London, UK.

^2^Kings Ophthalmology Research Unit, Kings’ College Hospital, London, UK.

^3^South East London Diabetic Eye Screening Programme, Guy’s and St Thomas’ Foundation Trust, London, UK.

^4^Department of Ophthalmology, Guy’s and St Thomas’ Foundation Trust, London, UK.

^5^Department of Diabetes, School of Life Course Sciences, King's College London, London, UK.

^6^Institute of Diabetes, Endocrinology and Obesity, King's Health Partners, London, UK.

^7^School of Biomedical Engineering & Imaging Sciences, King’s College London, London, UK.

*Correspondence: [p.nderitu@doctors.org.uk](mailto:p.nderitu@doctors.org.uk)

Contents

[Supplementary information 3](#_Toc106272274)

[Ground-truth definitions 3](#_Toc106272275)

[Model development 4](#_Toc106272276)

[Image pre-processing and training augmentations 4](#_Toc106272277)

[Model architecture 4](#_Toc106272278)

[Model training and tuning methodology 5](#_Toc106272279)

[Table S2. Image specifications 7](#_Toc106272280)

[Table S3. External test dataset characteristics 8](#_Toc106272281)

[Table S4. Multi-output model internal test set performance stratified by demographic characteristics 9](#_Toc106272282)

[Table S5. Multi-output model laterality and gradability internal test set performance stratified by retinal field 10](#_Toc106272283)

[Table S6. Diabetic retinopathy screening grading definitions 11](#_Toc106272284)

[Figure S5: External test dataset single-output model pixel attribution maps 12](#_Toc106272285)

[Figure S6: Custom labelling app 13](#_Toc106272286)

[Figure S7: Example images and labels 14](#_Toc106272287)

[Figure S8: Multi-output model architecture 15](#_Toc106272288)

[References 16](#_Toc106272289)

# Supplementary information

## Ground-truth definitions

**Laterality:** Images were labelled left or right as identifiable for both anterior segment and retinal images. Where it was not possible to determine the laterality, the image laterality label was unidentifiable. Therefore, possible laterality classes were ***right, left*** or ***unidentifiable***.

**Retinal Presence:** Images from any retinal view were labelled as ***retinal*** otherwise they were ***non-retinal*** (*e.g., anterior segment or miscellaneous images*). Miscellaneous images were of the room/background/blank with no discernible retinal or anterior eye features.

**Retinal Field:** Images were labelled as macula if the fovea was located >2 disc diameters from the image edge. If the fovea was obscured, the foveal position was estimated using a point 2.5 disc diameters from the optic disc temporally, starting at the inferior margin of the optic disc. Images were labelled as nasal if the visible optic disc was >2 disc diameters from the superior and inferior edges of the image edge and either >2 disc diameters from the medial image edge or the optic disc was within the temporal image edge (**Supplementary Fig. S7**). All other retinal images were labelled as other retinal field. Possible retinal field classes were ***macula, nasal*** or ***other retinal field***.

**Gradability:** The definition of gradability was in conformity with UK DR screening guidelines^1^ which also references the retinal field, therefore images were gradable if:

1. **Macular image**: The centre of fovea was >2 disc diameters from the image edge (*within the macula zone*) and fine vessels were visible within 1 disc diameter of the fovea.
2. **Nasal image**: The complete optic disc was >2 disc diameters from image edge (*within the gradable zone*) and fine vessels were visible on surface of disc (**Supplementary Fig. S7**).

Images from other retinal fields or nasal images where the optic disc was not within the gradable zone were labelled as ungradable. Possible gradability labels were either ***gradable*** or ***ungradable***.

## Model development

### Image pre-processing and training augmentations

Images were resized to 224x224x3 from their native resolution using bilinear interpolation. For models trained to classify retinal field or gradability, left eye images were horizontally flipped to a right orientation to reduce inter-image variance, otherwise images were loaded in their native orientation for laterality and retinal presence models. During inference, the laterality model would be applied first to classify laterality prior to the use of field and gradability models. Sequential random augmentations of hue (+/- 0.025), contrast (-0.25, +0.5), saturation (-0.25, +0.5), brightness (+/- 0.15) and vertical flip were applied during model training with each augmentation having a probability of application of 0.5^2^.

### Model architecture

EfficientNet has recently been shown to achieve state-of the-art accuracy and efficiency with 6x faster inference speed at 8x less computational cost and with good transfer capability^3^. EfficientNet has incorporated image rescaling and per channel normalisation layers $\left( \frac{x-\mu}{\sigma} \right)$, where x is the input pixel value, μ is the image per channel mean value and σ is the standard deviation value. ‘ImageNet’ based per channel (*red, green, blue*) mean and standard deviation constants μ = [0.485, 0.456, 0.406] and σ = [0.229, 0.224, 0.225] were used for image normalisation. EfficientNet-V1-B0 with ImageNet weight initialisation was used as the feature extractor (*encoder*). This encoder model was adjoined to an untrained, randomly initialised classification network consisting of 3 x 3 depth-wise separable 2D convolution^4^, batch normalisation^5^ and flattened feature layer prior to a final dropout layer^6^ and classification node. Multi-output models shared the final flattened feature and dropout layers (**Supplementary** **Fig. S8**).

### Model training and tuning methodology

RMSProp with a momentum of 0.9 was used to minimise the mean proportional class weighted (1) binary or categorical cross entropy (CE) loss (2) for binary or multiclass outputs respectively. Proportional class weighting was applied to single-output models only. For multi-output models, respective losses were weighted 1:1.

1. $Propotional class weight =\frac{1}{number of class samples} x \frac{train dataset samples}{number of classes}$
2. $Weighted CE = -\frac{1}{N}\sum_{i=1}^{N} \sum_{j=1}^{K} {w_{j}*t}_{i,j}\log\left( p_{i,j} \right)$, where $N$ = total samples, $K$ = number of classes, $w_{j}$ = proportional class weight, $t_{i,j}$ is the ground-truth and $p_{i,j}$ is the prediction.

A random search of the learning rate $\left[ 1e^{-4}, 1e^{-2} \right]$, dropout $\left[ 0.2, 0.5 \right]$ and weight regularisation $\left[ 1e^{-4}, 1e^{-2} \right]$ hyperparameters (*uniform distribution*) was performed with the aim of selecting the parameters with the maximum AUROC (*single-output models*) or minimum loss (*multi-output models*) on the internal validation set. Ten random search iterations each with a maximum of 10 epochs were performed.Figure S1: Routine digital diabetic eye screening and internal dataset site distributions


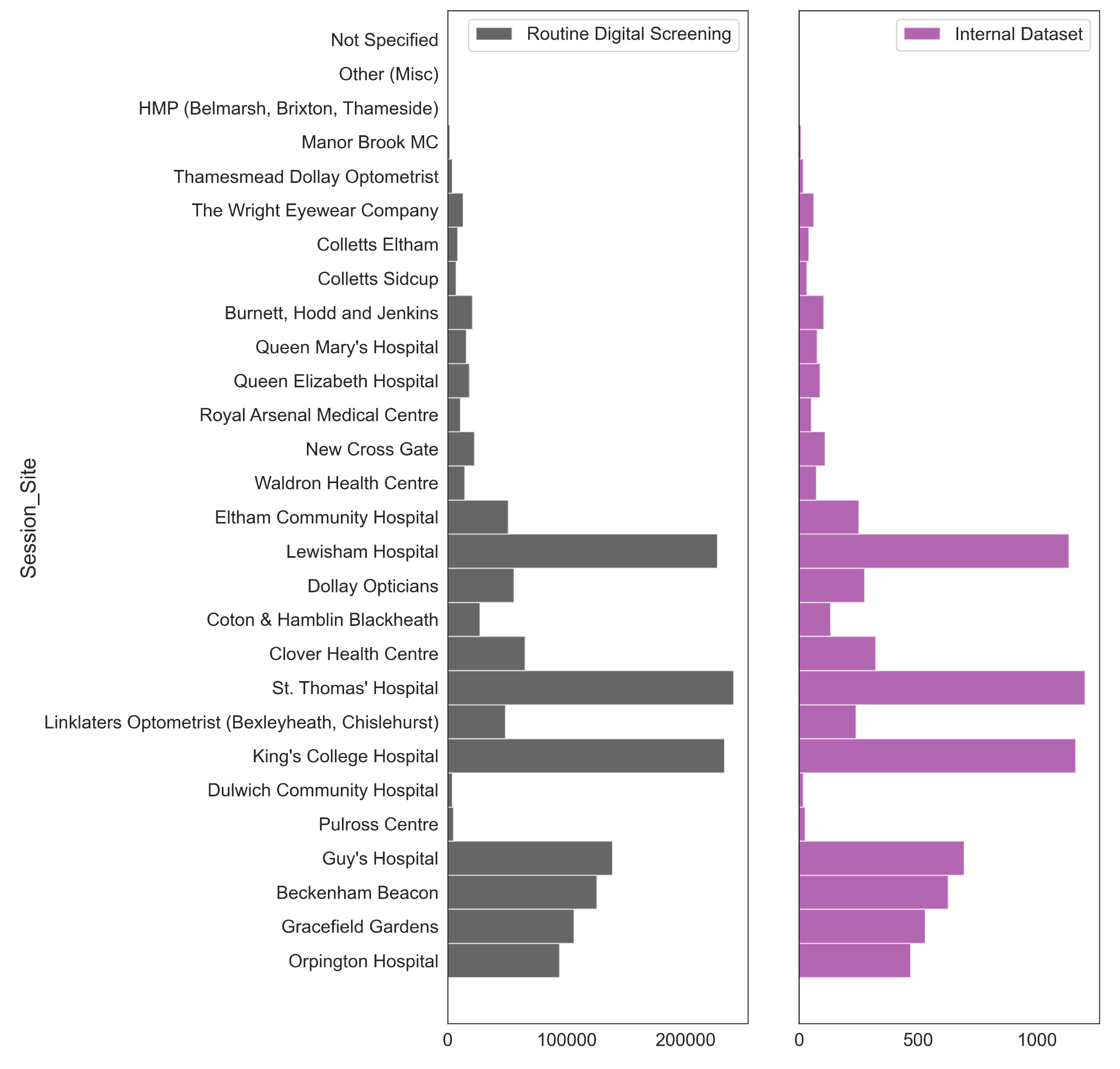


Count of images from routine digital diabetic eye screening and representative sample that formed the internal dataset showing proportional representation of the 27 screening sites.

# Table S2. Image specifications

| Variable | Internal Dataset | External Test Dataset | |
| --- | --- | --- | --- |
|  | **South-East London Diabetic Eye Screening Programme**  N = 7,743 | **UBIRIS**  N = 42 | **Universidad Nacional de Asunción**  N = 1,437 |
| Dataset Version | *Not applicable* | UBIPr | V0.3 |
| Country [City] | United Kingdom [London] | Portugal [Covilha] | Paraguay [Asunción] |
| Setting [Sites] | Routine Diabetic Eye Screening  [Hospital and Community Sites] | *Not reported* | Hospital  [Ophthalmology Department] |
| Capture Dates | Sept 2013 to Dec 2019 | *Not reported* | *Not reported* |
| Image Type | Colour | Colour | Colour |
| Mydriasis | Yes | No | Yes |
| Image Formats | jpg (84%), nef (16%) | jpg (100%) | jpg (100%) |
| Imaged Field | 45° | Periocular | 45° |
| Image Resolution | [1960 x 1934] to [6000 x 4000] | [593 x 409] to [1494 x 1031] | 2124 x 2056 |
| Cameras | Nikon [D70S, D80, D90, D7000, D7100, D7200] paired with Topcon [NW6, NW8] | Canon EOS 5D | Zeiss Visucam 500 |
| Download Link | *Not applicable* | <http://iris.di.ubi.pt/ubipr.html> | <https://zenodo.org/record/4891308#.YXgLsp7ML-g> |

**N:** Images

# Table S3. External test dataset characteristics

| Variable | | Laterality and Retinal Presence Dataset  N = 1,479 | Non-Retinal & Unidentifiable Laterality Images Removed (N = 52) | Retinal Field and Gradability Dataset  N = 1,427 |
| --- | --- | --- | --- | --- |
|  |  | N (%) |  | N (%) |
| DR Grade^a^ | No STDR | 717 (48.5) |  | 717 (50.2) |
|  | STDR | 720 (48.7) |  | 710 (49.8) |
|  | Not Applicable | 42 (2.8) |  | 0 (0.0) |
| Laterality | Right | 727 (49.2) |  | 711 (49.8) |
|  | Left | 742 (50.2) |  | 716 (50.2) |
|  | Unidentifiable | 10 (0.7) |  | - |
| Retinal Presence | Non-Retinal | 42 (2.8) |  | - |
|  | Retinal | 1,437 (97.2) |  | 1,427 (100) |
| Retinal Field | Macula | - |  | 1,059 (74.2) |
|  | Nasal | - |  | 157 (11.0) |
|  | Other Retinal Field | - |  | 211 (14.8) |
| Gradability | Ungradable | - |  | 408 (28.6) |
|  | Gradable | - |  | 1,019 (71.4) |

^a^STDR ophthalmologist derived ground truth, moderate or worse DR or maculopathy. **N:** Images, **SD**: Standard deviation, **DR**: Diabetic retinopathy, **STDR**: Sight-threatening diabetic retinopathy.

# Table S4. Multi-output model internal test set performance stratified by demographic characteristics

| Variable | | | Laterality and Retinal Presence Model | | | | | Non-Retinal & Unidentifiable Laterality Images Removed | Retinal Field and Gradability Model | | | | |
| --- | --- | --- | --- | --- | --- | --- | --- | --- | --- | --- | --- | --- | --- |
|  |  |  | **Test**  **size** | **Laterality^a^** | | **Retinal Presence^b^** | |  | **Test**  **size** | **Retinal Field^a^** | | **Gradability^b^** | |
|  |  |  |  | **Sensitivity** | **Specificity** | **Sensitivity** | **Specificity** |  |  | **Sensitivity** | **Specificity** | **Sensitivity** | **Specificity** |
|  |  |  |  | (95%CI) | | | |  |  | (95%CI) | | | |
| Age Groups (years) | | 12-39y | 98 | 0.97  (0.88-1.00) | 0.97  (0.87-1.00) | 1.00  (0.96-1.00) | 1.00  (0.29-1.00) |  | 103 | 1.00  (0.96-1.00) | 1.00  (0.98-1.00) | 0.99  (0.94-1.00) | 0.93  (0.66-1.00) |
|  |  | 40-49y | 174 | 0.97  (0.93-0.99) | 0.99  (0.97-1.00) | 1.00  (0.98-1.00) | 0.80  (0.28-0.99) |  | 179 | 0.97  (0.94-0.99) | 0.99  (0.97-1.00) | 0.99  (0.95-1.00) | 0.90  (0.73-0.98) |
|  |  | 50-59y | 366 | 0.97  (0.94-0.99) | 0.99  (0.96-1.00) | 1.00  (0.99-1.00) | 1.00  (0.72-1.00) |  | 337 | 0.99  (0.97-1.00) | 0.99  (0.98-1.00) | 0.98  (0.96-0.99) | 0.82  (0.68-0.91) |
|  |  | 60-69y | 386 | 0.97  (0.95-0.98) | 0.98  (0.97-0.99) | 1.00  (0.99-1.00) | 0.96  (0.80-1.00) |  | 333 | 0.98  (0.96-0.99) | 0.99  (0.98-1.00) | 0.98  (0.96-0.99) | 0.90  (0.76-0.97) |
|  |  | 70-79y | 325 | 0.94  (0.89-0.97) | 0.99  (0.96-1.00) | 1.00  (0.98-1.00) | 1.00  (0.85-1.00) |  | 311 | 0.97  (0.95-0.99) | 0.99  (0.97-0.99) | 0.96  (0.93-0.98) | 0.90  (0.80-0.96) |
|  |  | ≥80y | 192 | 0.88  (0.79-0.94) | 0.94  (0.88-0.98) | 1.00  (0.98-1.00) | 1.00  (0.80-1.00) |  | 203 | 0.97  (0.93-0.99) | 0.98  (0.96-0.99) | 0.90  (0.84-0.94) | 0.78  (0.64-0.88) |
| Sex | Female | | 709 | 0.96  (0.94-0.97) | 0.98  (0.97-0.99) | 1.00  (0.99-1.00) | 0.98  (0.88-1.00) |  | 659 | 0.98  (0.97-0.99) | 0.99  (0.98-0.99) | 0.96  (0.94-0.97) | 0.86  (0.78-0.92) |
|  | Male | | 832 | 0.97  (0.95-0.98) | 0.98  (0.98-0.99) | 1.00  (1.00-1.00) | 0.98  (0.87-1.00) |  | 807 | 0.98  (0.96-0.98) | 0.99  (0.98-0.99) | 0.98  (0.96-0.99) | 0.86  (0.79-0.91) |
| Ethnicity | White | | 772 | 0.97  (0.95-0.98) | 0.98  (0.98-0.99) | 1.00  (0.99-1.00) | 0.97  (0.86-1.00) |  | 749 | 0.98  (0.97-0.99) | 0.99  (0.98-0.99) | 0.97  (0.96-0.99) | 0.89  (0.82-0.93) |
|  | Black | | 462 | 0.94  (0.90-0.97) | 0.98  (0.95-0.99) | 1.00  (0.99-1.00) | 1 .00  (0.88-1.00) |  | 428 | 0.97  (0.95-0.99) | 0.99  (0.98-0.99) | 0.97  (0.94-0.98) | 0.80  (0.70-0.89) |
|  | South Asian^c^ | | 99 | 0.94  (0.85-0.99) | 0.98  (0.88-1.00) | 1.00  (0.96-1.00) | 1.00  (0.48-1.00) |  | 96 | 0.97  (0.91-0.99) | 0.98  (0.96-1.00) | 0.97  (0.91-1.00) | 0.89  (0.67-0.99) |
|  | Other Asian^d^ | | 103 | 0.95  (0.87-0.99) | 0.97  (0.87-1.00) | 1.00  (0.96-1.00) | 1.00  (0.59-1.00) |  | 97 | 0.98  (0.93-1.00) | 0.99  (0.98-1.00) | 0.98  (0.92-1.00) | 0.91  (0.59-1.00) |
|  | Mixed | | 37 | 0.95  (0.82-0.99) | 0.97  (0.91-1.00) | 1.00  (0.90-1.00) | 0.50  (0.01-0.99) |  | 35 | 0.97  (0.85-1.00) | 0.99  (0.92-1.00) | 0.90  (0.73-0.98) | 0.67  (0.22-0.96) |
|  | Other^e^ | | 36 | 1.00  (0.82-1.00) | 0.94  (0.71-1.00) | 1.00  (0.89-1.00) | 1.00  (0.29-1.00) |  | 32 | 0.97  (0.84-1.00) | 0.98  (0.92-1.00) | 0.96  (0.78-1.00) | 0.89  (0.52-1.00) |
|  | Not Specified | | 32 | 1.00  (0.81-1.00) | 1.00  (0.77-1.00) | 1.00  (0.89-1.00) | * |  | 29 | 1.00  (0.88-1.00) | 1.00  (0.94-1.00) | 0.96  (0.79-1.00) | 1.00  (0.48-1.00) |
| All | | | 1,541 | 0.96  (0.95-0.97) | 0.98  (0.98-0.99) | 1.00  (1.00-1.00) | 0.98  (0.92-1.00) |  | 1,466 | 0.98  (0.97-0.98) | 0.99  (0.98-0.99) | 0.97  (0.96-0.98) | 0.86  (0.81-0.90) |

*No specificity as all cases were positive. ^a^Model operating point = argmax, ^b^Model operating point threshold = 0.5, ^c^Includes Indian, Bangladeshi, and Pakistani ethnic backgrounds, ^d^Includes any other asian background or Chinese, ^e^Includes any other ethnic group or Arab, **CI**: Confidence interval (*Clopper-Pearson exact method^7^*).

# Table S5. Multi-output model laterality and gradability internal test set performance stratified by retinal field

| Variable | | Laterality and Retinal Presence Model | | | Non-Retinal & Unidentifiable Laterality Images Removed | Retinal Field and Gradability Model | | |
| --- | --- | --- | --- | --- | --- | --- | --- | --- |
|  |  | **Test**  **size** | **Laterality^a^** | |  | **Test**  **size** | **Gradability^b^** | |
|  |  |  | **Sensitivity** | **Specificity** |  |  | **Sensitivity** | **Specificity** |
|  |  |  | (95%CI) | |  |  | (95%CI) | |
| Retinal Field | Macula | 673 | 0.99  (0.98-0.99) | 0.99  (0.97-0.99) |  | 673 | 0.96  (0.97-0.96) | 0.76  (0.61-0.88) |
|  | Nasal | 662 | 0.97  (0.96-0.98) | 0.97  (0.96-0.98) |  | 653 | 0.97  (0.96-0.99) | 0.69  (0.58-0.80) |
|  | ORF | 122 | 0.93  (0.87-0.97) | 0.93  (0.87-0.97) |  | 140 | * | 0.98  (0.94-1.00) |

*Images from other retinal fields (ORF) are ungradable by definition, therefore, because all of these cases are negative, only specificity can be computed. ^a^Model operating point = argmax, ^b^Model operating point threshold = 0.5, **CI**: Confidence interval (*Clopper-Pearson exact method^7^*), **ORF**: Other retinal field.

# Table S6. Diabetic retinopathy screening grading definitions

| Grade | Description | Criteria |
| --- | --- | --- |
| Retinopathy | | |
| R0 | ***No DR*** | - No diabetic retinopathy retinal lesions |
| R1 | ***Mild-moderate non-proliferative DR*** | - Presence of microaneurysms or retinal haemorrhages - Exudates or cotton wool spots in the presence of DR features |
| R2* | ***Moderate-severe non-proliferative DR*** | - Presence of multiple blot haemorrhages, venous beading or intraretinal microvascular abnormalities |
| R3A* | ***Proliferative DR*** | - New features of proliferative disease, namely, new vessels at the disk or elsewhere, preretinal/vitreous haemorrhage, new preretinal fibrosis or new tractional retinal detachment - Previous treatment for proliferative DR that has not been deemed stable by the treating ophthalmologist - New features indicating reactivation of proliferation, or potentially sight threatening change from fibrous proliferation with respect to previously reference images |
| R3S | ***Stable-treated proliferative DR*** | - Evidence of peripheral retinal laser treatment and stable retina with respect to reference images taken at or shortly after discharge from the hospital eye service. |
| Maculopathy | | |
| M0 | ***No referable diabetic maculopathy*** | - *Referable diabetic maculopathy criteria not met* |
| M1* | ***Referable diabetic maculopathy*** | - Exudate ≤1 disc diameter from the fovea - ≥1/2 a disc area of exudates within the macula - Microaneurysm ≤1 disc diameter from the fovea AND visual acuity ≤6/12 |

***Sight-threatening diabetic retinopathy** as per UK national screening committee definitions^8^, **DR**: Diabetic retinopathy.

# Figure S5: External test dataset single-output model pixel attribution maps


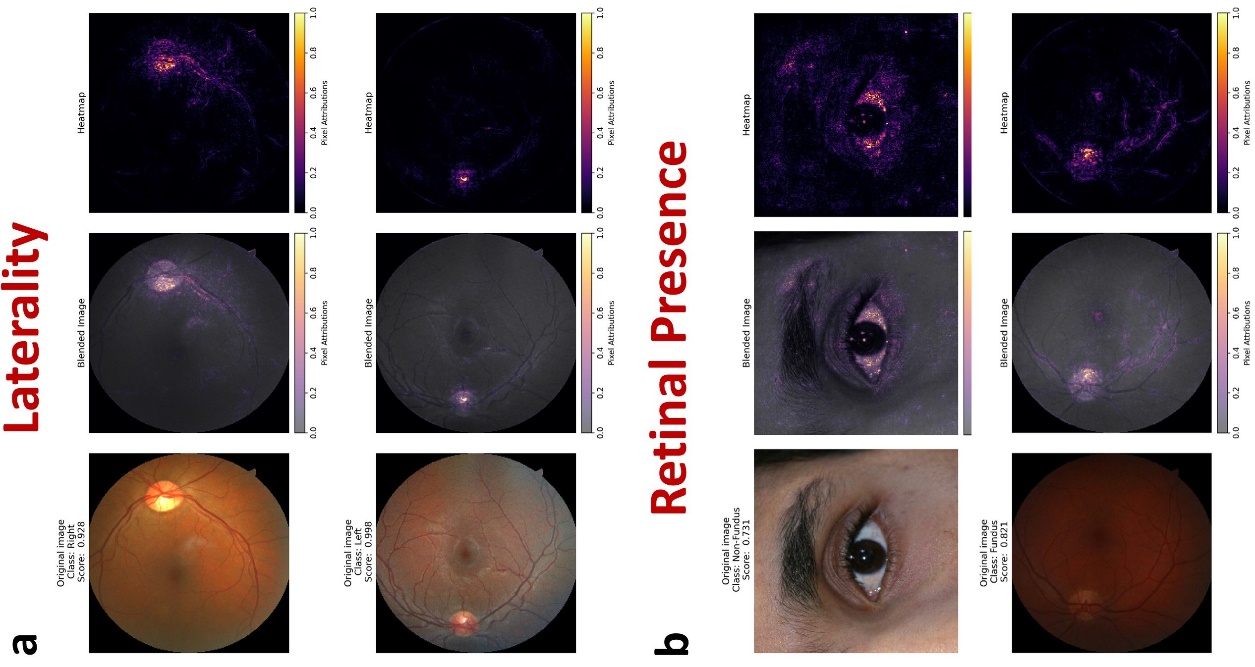


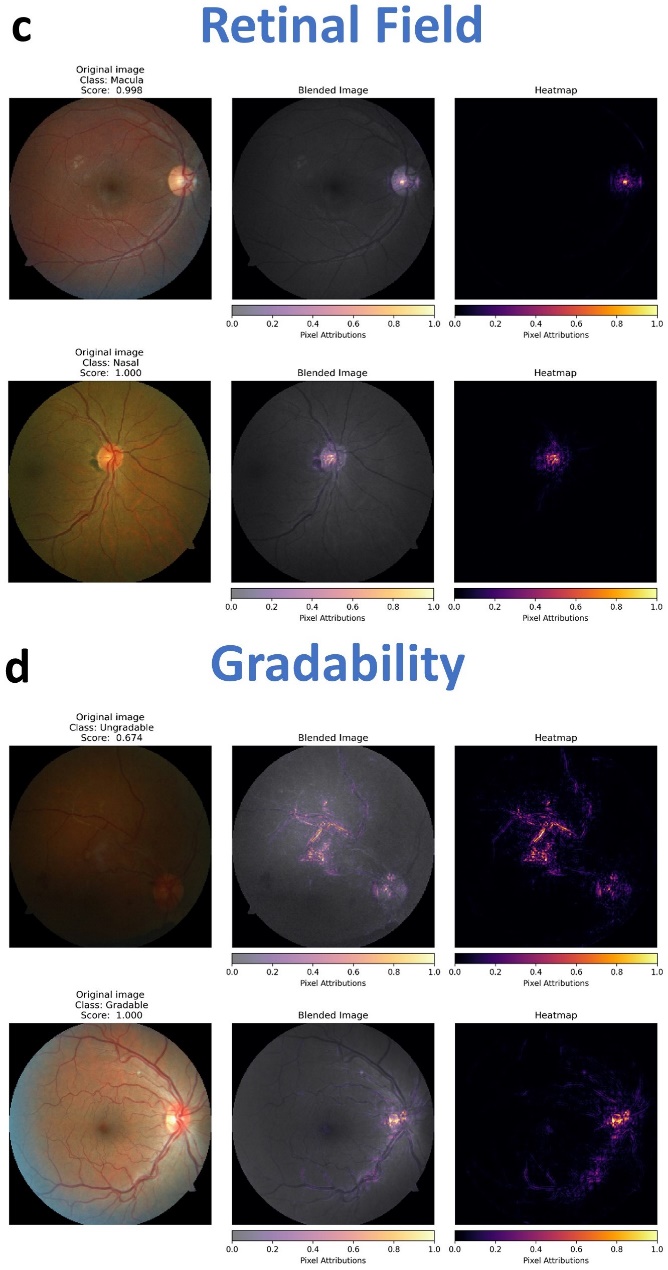


**Integrated gradients pixel attributions**: All models highlight the optic cup/disc within retinal images, especially model c. Models a, b and d also highlight the retinal vessels to varying degrees. Model b (*non-retinal image*) highlights the conjunctival vessels and corneal reflections. Model d (*ungradable image*) highlights the superotemporal retinal vessels to identify the displaced retinal image as ungradable. Model attributions relative to the true positive class in each example.

# Figure S6: Custom labelling app


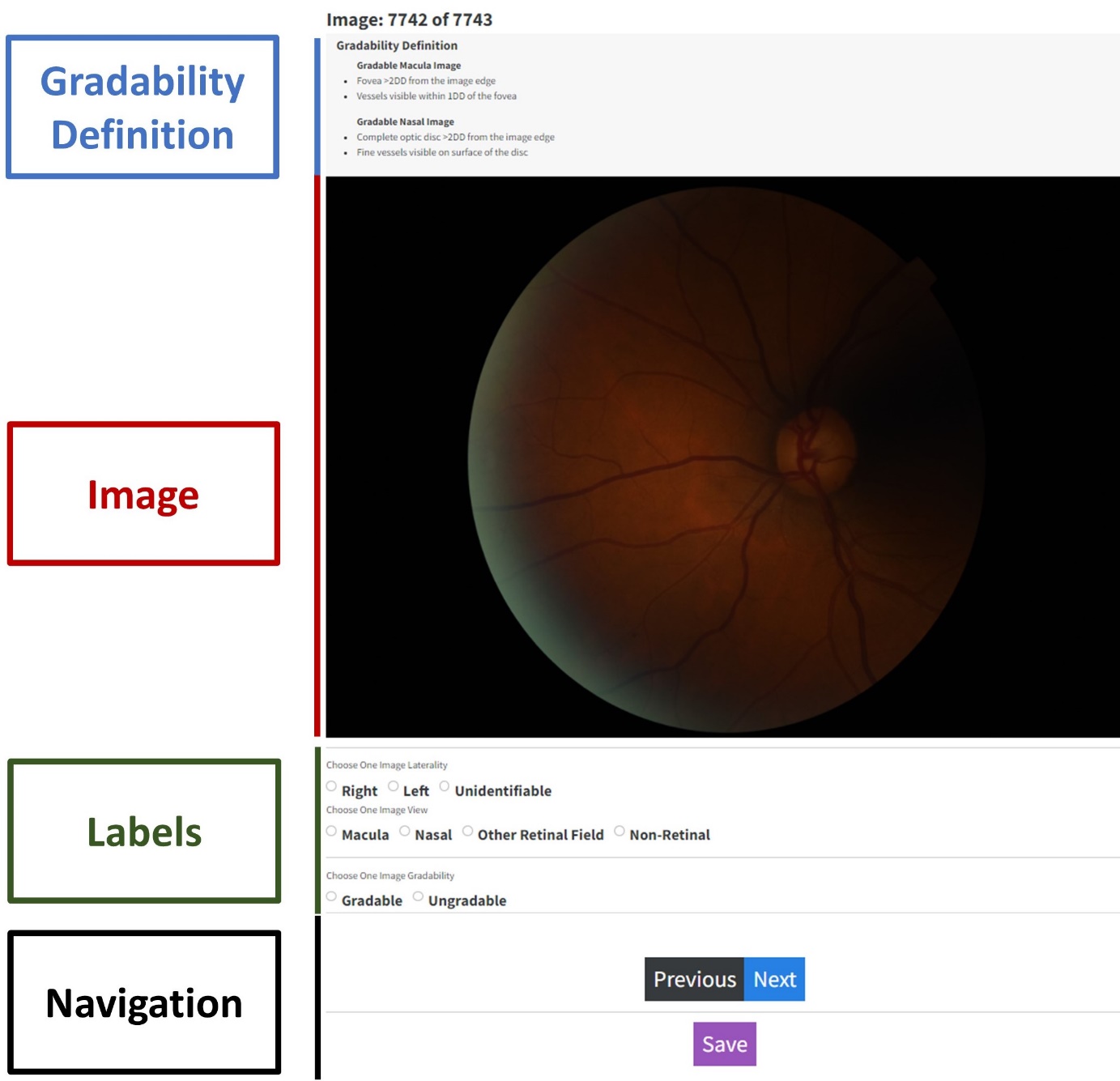


**Custom grading app:** App made using the dash python library. UK national diabetic retinopathy screening gradability definition is displayed above every image. Images are loaded in a standardised size without additional pre-processing. Labels are designed with respect to the curation tasks, ensuring all sampled images could be categorised by laterality and retinal presence. Retinal images of known laterality (right or left) are also graded for retinal field and gradability. Radio buttons and error logic are used during grading to ensure complete and robust labelling.

# Figure S7: Example images and labels


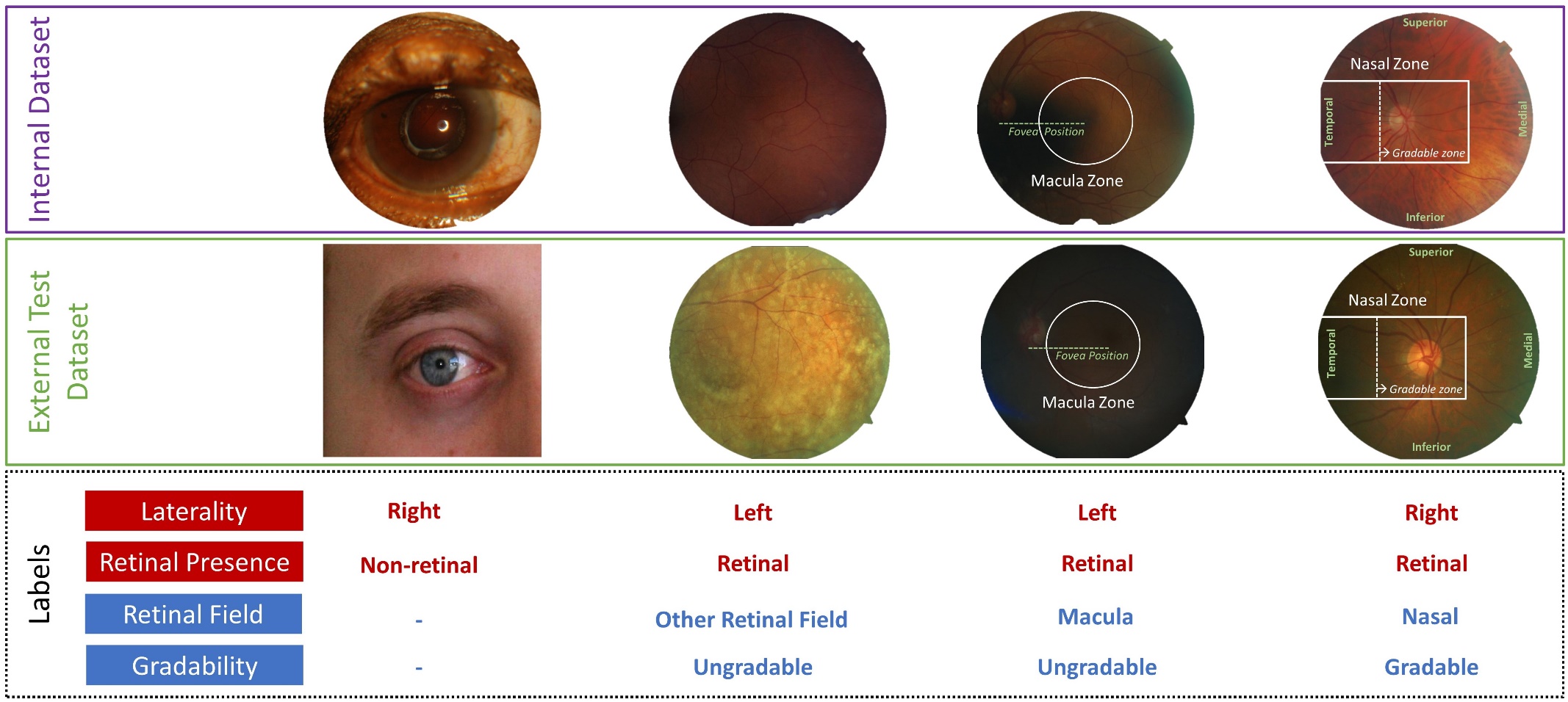


Examples of images and labels from the internal and external test dataset.

# Figure S8: Multi-output model architecture


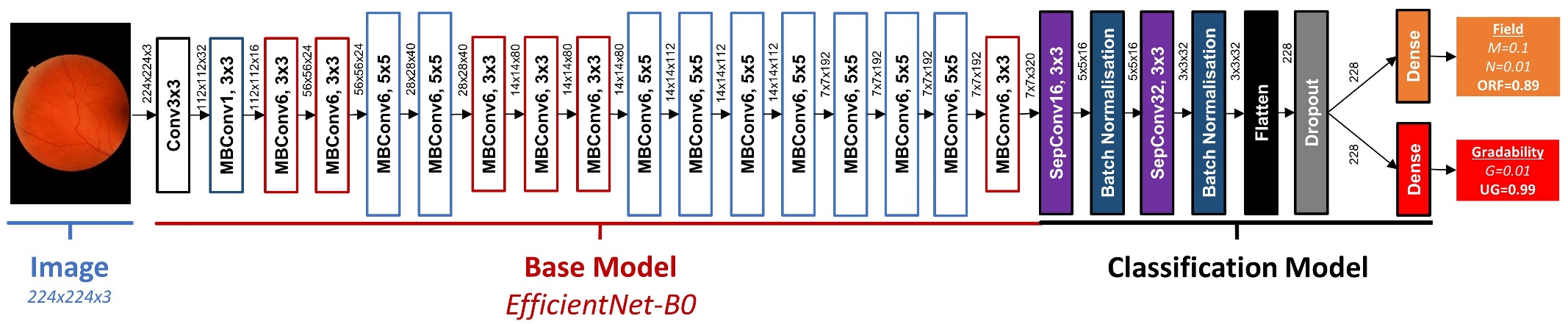


Retinal field and gradability multi-output model architecture. **M**: Macula, **N**: Nasal, **ORF**: Other retinal field, **G**: Gradable, **UG**: Ungradable.

# References

1 PHE. *Diabetic eye screening: guidance when adequate images cannot be taken*, <<https://www.gov.uk/government/publications/diabetic-eye-screening-pathway-for-images-and-where-images-cannot-be-taken/diabetic-eye-screening-guidance-when-adequate-images-cannot-be-taken>> (2021).

2 Agustin, T., Utami, E. & Fatta, H. A. in *2020 3rd International Conference on Information and Communications Technology (ICOIACT).* 83-88.

3 Mingxing, T. Q., V. L.;. EfficientNet: Rethinking Model Scaling for Convolutional Neural Networks. *arXiv*, 1-11 (2020).

4 Tan, M. & Le, Q. V. EfficientNet: Rethinking Model Scaling for Convolutional Neural Networks. arXiv:1905.11946 (2019). <<https://ui.adsabs.harvard.edu/abs/2019arXiv190511946T>>.

5 Ioffe, S. & Szegedy, C. Batch Normalization: Accelerating Deep Network Training by Reducing Internal Covariate Shift. arXiv:1502.03167 (2015). <<https://ui.adsabs.harvard.edu/abs/2015arXiv150203167I>>.

6 Labach, A., Salehinejad, H. & Valaee, S. Survey of Dropout Methods for Deep Neural Networks. *ArXiv* **abs/1904.13310** (2019).

7 Clopper, C. J. & Pearson, E. S. THE USE OF CONFIDENCE OR FIDUCIAL LIMITS ILLUSTRATED IN THE CASE OF THE BINOMIAL. *Biometrika* **26**, 404-413, doi:10.1093/biomet/26.4.404 (1934).

8 PHE. *NHS Diabetic Eye Screening Programme: Grading definitions for referable disease*, <<https://www.gov.uk/government/publications/diabetic-eye-screening-retinal-image-grading-criteria/nhs-diabetic-eye-screening-programme-grading-definitions-for-referable-disease>> (2021).
